# Longitudinal Mode Number Estimation of External Cavity Diode Laser Using Dual Periodic Grating for Optical Profiler System

**DOI:** 10.3390/s24123821

**Published:** 2024-06-13

**Authors:** Masaki Michihata, Shuhei Goda, Shuzo Masui, Satoru Takahashi

**Affiliations:** Department of Precision Engineering, the University of Tokyo, 7-3-1 Hongo, Bunkyo, Tokyo 113-8656, Japan

**Keywords:** longitudinal mode, dual periodic grating, external cavity diode laser

## Abstract

The concept of an optical profiler based on optical resonance was proposed, highlighting the initial requirements for mode number estimation. We proposed a method for estimating the longitudinal mode number of a laser propagating in an external cavity diode laser with high accuracy, utilizing dual-periodic diffraction gratings. These gratings were fabricated using interference lithography. To estimate the mode number, the wavelengths of two different modes are compared. Therefore, the greater the difference between the wavelengths, the higher the accuracy of the mode number determination. While the mode number difference was approximately 35 when using a conventional diffraction grating, this could be increased by a factor of 20 to around 700 using the dual-periodic grating. The relative accuracy achieved was 1.4 × 10^−5^.

## 1. Introduction

Recent advancements in production technology integrating digital technologies such as Digital Twin and Industry 4.0 necessitate higher accuracy measurement technology to align scales in the virtual and real domains [1,2]. Alongside traditional three-dimensional form metrology like coordinate measuring machines (CMM) [3,4,5], there is a rising demand for rapid and precise shape measurement in factory environments, particularly advanced on-machine and in-process measurements [6,7,8]. Optical profilers capable of capturing the 2.5-dimensional shape of objects are in high demand, requiring enhanced measurement speed, high lateral and optical-axis resolution, and extended working distances. Various methods have been proposed to address these requirements [9,10,11,12,13,14,15,16,17,18,19,20].

The most common optical method for displacement measurement is the triangulation method [10,11]. This method offers simple measurement principles and system configurations, allowing for fast measurements. However, it has its disadvantages. To achieve higher resolution, the working distance needs to be narrow. Additionally, structures with a high aspect ratio are challenging to measure due to blocked response signals, such as reflected and scattered light, by the measuring object itself. Chromatic confocal sensors represent another commonly used method [12,13,14]. These sensors use chromatic aberration, leading to a trade-off between resolution and measurement range. While the resolution can reach as high as a few nanometers, the measurement range is limited to below 1 mm. Furthermore, the linearity of the signal depends on the optical system, necessitating careful calibration across the entire measurement range for high accuracy. Interferometer-based methods have also been proposed [15,16,17,18]. In principle, interferometers offer high accuracy and a wide working distance. Utilizing multiple wavelengths can help avoid phase jumps, a critical issue in interferometry. However, in practice, measurement accuracy is largely dependent on the quality of the light source. Specifically, wavelength fluctuations significantly affect the measurement accuracy. Additionally, the high-resolution reference plane can experience position drift, impacting the measurement uncertainty. As interferometry is essentially a displacement measurement method, a stable environment is crucial when measuring absolute dimensions of shapes, including steps. Techniques employing optical frequency combs [19] and holographic microscopes [20] also demonstrate impressive performance, such as the ability to measure surfaces with high accuracy and speed. Nonetheless, their adoption in factories is challenging due to system costs, system complexity, and other factors.

Therefore, this study focuses on optical profilers based on optical resonance. While optical resonance typically requires precise alignment, such as in laser cavities [21], it is not commonly used for free-form metrology. However, it can achieve a high Q-value, leading to high measurement resolution when used for measurement purposes [22,23,24,25]. To enhance alignment robustness, an external resonator construction can be introduced [26,27]. By configuring a cavity with the measurement target surface and measuring the cavity length, the displacement of the surface can be determined. Since the cavity length is measured, phase jumps are no longer an issue. Additionally, using optical frequency as the measurement quantity ensures metrological traceability. Nevertheless, achieving an accurate measurement system requires addressing the precise estimation of the cavity length [28]. The cavity length estimation relies on the longitudinal mode number and the resonance wavelength. While the resonance wavelength can be directly measured, the longitudinal mode number needs to be estimated. The accuracy of this estimation directly influences the measurement accuracy. In this study, we propose a method for estimating the longitudinal mode number for the external cavity diode laser and discuss its accuracy.

## 2. Concept of Optical Profiler Based on External Cavity Diode Laser

**Figure 1:**
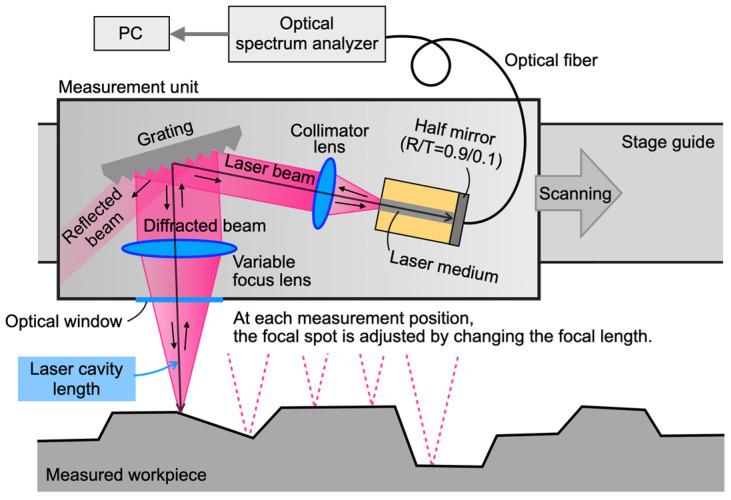
Conceptual sketch of the external cavity diode laser-based optical profiler system.

**Figure 2:**
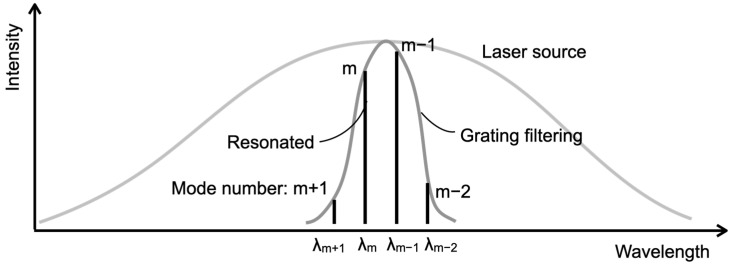
Optical spectrum in the external cavity diode laser.

The concept of the external cavity diode laser (ECDL)-based optical profiler proposed in this study is illustrated in Figure 1. The profiler uses a semiconductor gain half-chip as the optical pumping medium. One side features an anti-reflection coating, while the other side has a half-mirror with 90% reflectivity. The light emitted from the medium is collimated through a lens and directed towards a diffraction grating. Here, the first-order diffracted light is focused onto the measuring surface. The light emitted from the source medium covers a wide wavelength range of several tens of nanometers but is dispersed by the diffraction grating. Consequently, only the diffracted light wavelengths perpendicular to the measurement plane remain in the resonator. Within the wavelength band filtered by the grating, the wavelengths that resonate between the measurement surface and the laser gain chip’s mirror are isolated. This results in discrete resonant wavelengths, as depicted in Figure 2. As the measuring surface displaces, a variable focus lens is employed to maintain focus on the surface. The reflected light from the measurement surface returns to the laser gain chip, leading to a resonance state. The wavelengths of this resonance are determined by the distance to the measurement surface, namely the cavity length. The light transmitted through the laser gain chip undergoes spectral analysis using an optical spectrum analyzer. This analysis identifies the resonance wavelengths, enabling the determination of the cavity length and resonance wavelength. The cavity length can be calculated by multiplying the resonant wavelength by the corresponding longitudinal mode number, indicating the position of the measurement surface. Since this is a point measurement, it can be performed through either optical or mechanical scanning.

Here, determining the mode numbers from multiple resonance wavelengths is crucial, as the resonant wavelengths are obtained through the measurement. If these mode numbers can be accurately determined, the method can serve as a reliable measurement technique.

## 3. Principle of Longitudinal Mode Number Estimation of External Cavity Diode Laser Using Dual Periodic Grating

### 3.1. Fundamental Theory of Longitudinal Mode Number Estimation

The wavelength at which resonance occurs is determined by the resonance conditions. These conditions depend on factors such as the cavity length and the refractive index of the materials involved.

(1) $$2(n_{air}L+n_{m}^{LD}l)=m\times \lambda_{m}$$

where m is the longitudinal mode number, which is an integer; λ_m_ is the resonant wavelength of the longitudinal mode number m; L is the distance between the laser medium surface and the measured surface; l is the length of the laser medium; *n_air_* is the refractive index of air. *n_m_^LD^* is the refractive index of the laser medium. From this equation, the longitudinal mode number can be estimated by measuring two different modes, that is, m and m+k. By using the equations for these two modes and removing the distance to the surface, L, the longitudinal mode number, m, can be expressed by the following equation.

(2) $$m=\frac{2l\left(n_{m}^{LD}-n_{m+k}^{LD}\right)+k\lambda_{m+k}}{\lambda_{m}-\lambda_{m+k}}$$

Here, k indicates the difference between the longitudinal mode numbers of the two resonant wavelengths. Since the refractive index and tip length of the laser medium are known, the mode number, m, can be determined by measuring the resonance wavelengths. Using Equation (1), these values can then be used to calculate the cavity length, L, excluding the tip length.

### 3.2. Uncertainty of Longitudinal Mode Number Estimation

The longitudinal mode number is determined from two measured resonance wavelengths. This section discusses the variations in the estimation of the longitudinal mode number when the measurement of resonance wavelengths includes variations. The impact of wavelength measurement errors was investigated by varying the mode number difference, k, between the two wavelengths used for mode number estimation. Specifically, the estimation error for the mode number is calculated using Equation (2). The maximum amount of the estimated mode number deviation Δm is found when the measured wavelengths λ_m_ and λ_m+k_ each contain an error of Δλ. Here, *l* = 1 mm, L = 19 mm, and λ_m_ was set to 1550 nm. λ_m+k_ was calculated using k and λ_m_. Thus, Δm was calculated using Equation (3). The difference between *n_m_^LD^* and *n_m+k_^LD^* was ignored at this time because it is small compared to kλ. The mode number estimated error was calculated for dλ = 10 pm, dλ = 1 pm, dλ = 0.1 pm, and dλ = 0.01 pm, respectively.

(3) $$m+\Delta m=\frac{2l\left(n_{m}^{LD}-n_{m+k}^{LD}\right)+k(\lambda_{m+k}-\Delta \lambda )}{\left(\lambda_{m}+\Delta \lambda )-(\lambda_{m+k}-\Delta \lambda \right)}$$

**Figure 3:**
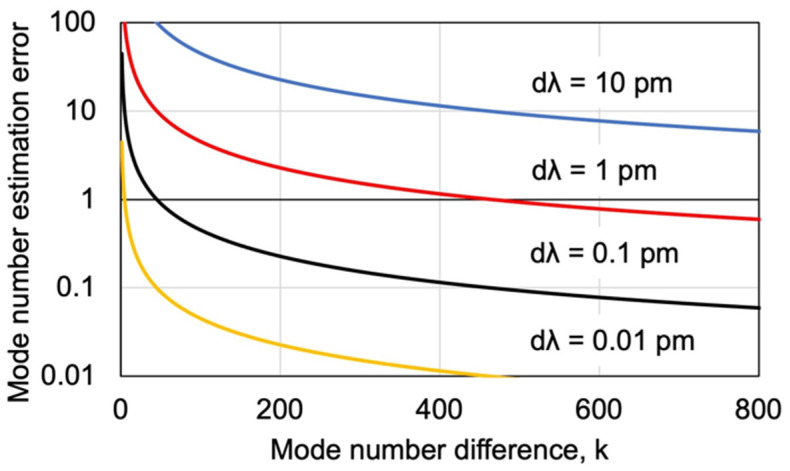
Mode number estimation error analysis.

Figure 3 illustrates the relationship between k, set on the horizontal axis, and the error in the estimation of the longitudinal mode number, Δm, set on the vertical axis. It is important to note that the vertical axis is logarithmic. Multiple modes were measured, and the mode number was estimated from the resonance wavelength of each. The study investigates how far apart these multiple modes should be for accurate estimation. Initially, it is evident that a smaller variation in wavelength measurement leads to more accurate mode number estimation. Additionally, increasing the difference in longitudinal mode number, k, reduces the error in its estimation. Notably, the accuracy of mode number estimation improves significantly when the difference in mode numbers reaches around 200. Therefore, for accurate mode number estimation, wavelengths as far apart as possible should be used.

In optical profiler applications for measuring step heights, absolute length measurement accuracy is crucial. This necessitates high accuracy in longitudinal mode number estimation. However, essentially, the absolute distance from the measuring surface to the reflecting mirror in the laser gain chip is not critical for the dimensional measurement of surface topography. From this perspective, the importance of accurate mode number measurement lies not in the precise measurement of the cavity length but in the precise understanding of changes in the cavity length. If the change in resonance wavelength with a change in cavity length ΔL is Δλ, then ΔL can be calculated using Equation (1) as

(4) $$∆L=\frac{m∆\lambda_{m}}{2n_{air}}$$

In other words, accurately measuring the mode number allows for the determination of the sensitivity coefficient between changes in the measurement surface and changes in the resonance wavelength.

### 3.3. Longitudinal Mode Number Estimation Using Dual Periodic Grating

**Figure 4:**
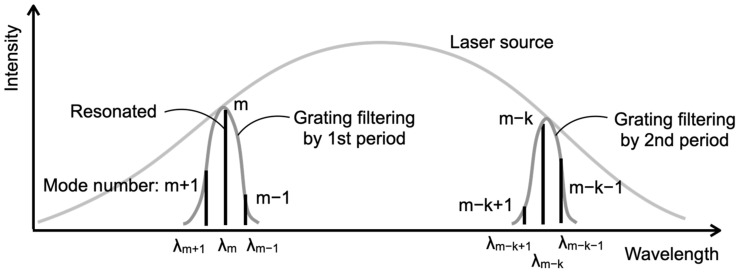
Optical spectrum in the external cavity diode laser with dual periodic grating.

To determine the mode number of a resonated mode, it is advantageous to use two modes that are as far apart in the wavelength band as possible. On the other hand, typically, when designing a laser, a single mode is preferred. Thus, a laser cavity is often designed for grazing incidence to extract a single mode when constructing an ECDL. In this study, however, we decrease the angle of incidence to make the laser multimode. The angle of incidence and the grating pitch significantly influence the extent to which resonance can be excited within a wavelength range of a few nanometers, resulting in the generation of multiple longitudinal modes. Despite selecting the two most distant modes within this narrow wavelength range, the mode number difference is typically only several tens. Consequently, a large mode number difference cannot be achieved, making accurate mode number determination challenging. To address this issue, a dual periodic grating [29,30,31] was employed as the diffraction grating. As the name suggests, this grating has a surface structure with two periods, enabling resonance in two different wavelength ranges simultaneously, as shown in Figure 4. As a result, modes with large wavelength differences can be selected. The greater the difference between the longitudinal mode numbers, k, of the two resonant wavelengths, the more accurate the mode estimation becomes. In subsequent experiments, we proposed a measurement method using a dual periodic grating and experimentally verified the accuracy of mode number estimation.

## 4. Experimental Setup

### 4.1. Optical System

**Figure 5:**
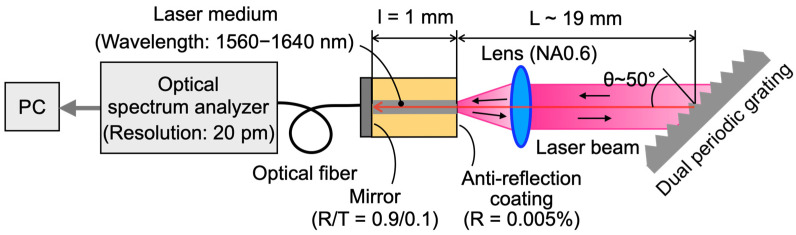
Illustration of experimental setup.

As depicted in Figure 1, the proposed measurement system is based on the Littman configuration. However, the primary aim of this paper is to investigate the effectiveness of the mode number estimation method using dual periodic grating. To validate the proposed method, the Littrow arrangement was employed, which is a simpler system. An illustration of the experimental setup based on the laser cavity with the Littrow configuration is shown in Figure 5. The experimental setup comprises a laser gain half-chip (Thorlabs SAE1550P2, bandwidth: 1560–1640 nm, AR-coated), a collimating lens (f = 2.97 mm, NA 0.6), and a dual periodic grating. The gain half-chip and collimating lens were fixed on the stage. The length of the laser medium was 1 mm. The light had an oscillation bandwidth of 1600 nm and was equipped with a thermoelectric cooler (TEC) temperature control mechanism to maintain a constant temperature of 25 °C. The laser aperture’s region emitting the laser beam was coated with an anti-reflective coating, where the reflectance was lower than 0.01%. The backside of the laser had a film with a reflectance of 90%. The lens was coated with an anti-reflection coating.

The resonant wavelength was measured from the back face of the LD using a single-mode fiber connected to an optical spectrum analyzer (Yokogawa AQ6370D, resolution 20 pm). This setup ensured stable wavelengths even when the diffraction grating was rotated. The incident angle of the diffraction grating was set to approximately 50 degrees, resulting in an extended cavity length of approximately 19 mm.

### 4.2. Dual Periodic Grating

The dual-periodic diffraction grating used was fabricated using photolithography. For detailed fabrication methods, refer to references [32,33]. Here is an overview of the fabrication process. In photolithography, light with a periodic intensity distribution was irradiated onto a photosensitive resin using a rotating Lloyd’s mirror optical system. Initially, a silicon substrate was hydrophobized. Subsequently, a positive photoresist (AZ electronic materials, AZP1350) was spin-coated onto the silicon, achieving a thickness of approximately 560 nm or thinner. The coated substrate was baked for 2 min. Next, the prepared silicon substrate was exposed to a standing wave using a He-Cd laser (IK3083R-D, λ_litho_ = 325 nm, Kinmon Koha Co., Ltd., Tokyo, Japan). The pitch, d, can be determined by the incident angle, θ, as d = λ_litho_/2sinθ. The first exposure was conducted with an incident angle, θ, which was approximately equal to around 9°. Subsequently, a second exposure was carried out with a rotation of 0.2°. After exposure, the substrate was developed using a developer. Finally, a layer of gold was deposited onto the diffraction grating to enhance its reflectance for the specific wavelength. The thickness of the gold film was approximately 100 nm.

The dual periodic grating played a crucial role in this study due to the difference in spatial frequency between the two pitches, which was set to a wavelength width of approximately 40 nm. Although the wavelength range of the LD spans 80 nm, from 1560 nm to 1640 nm, we focused on the relatively intense central 40 nm for this study. The mode number difference is theoretically sufficient within this 40 nm range, facilitating the verification of the proposed method. The conditions for satisfying the Littrow arrangement can be determined from the diffraction grating formula.

(5) $$d=\frac{\lambda }{2\sin\theta }$$

**Figure 6:**
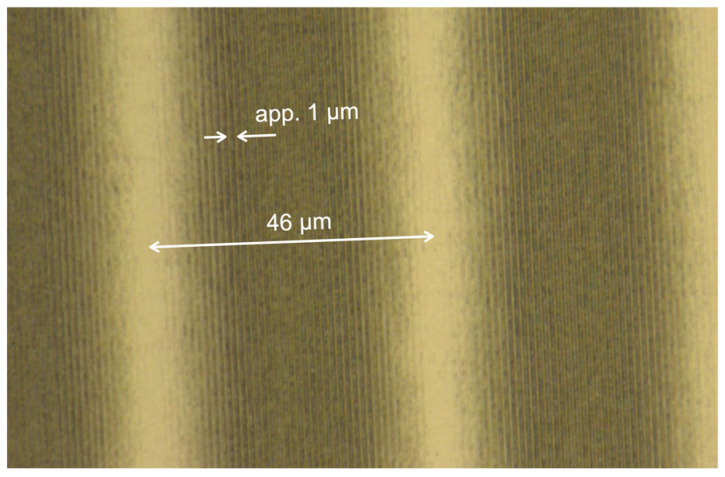
Microscopic image of a dual-periodic grating.

**Figure 7:**
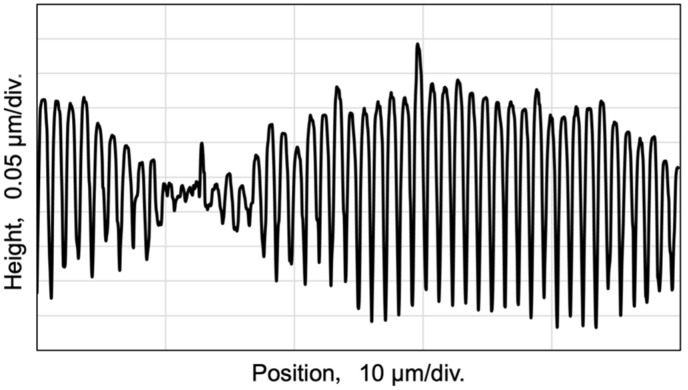
Height profile of a dual periodic grating measured by AFM.

In view of this, in order to make a difference of 40 nm resonance wavelength, a pitch difference of 26 nm was required, which corresponded to a spatial frequency difference of 0.021 µm^−1^. The fabricated grating was observed using optical microscopy (Figure 6). The pith of the grating was confirmed using atomic force microscopy (AFM). Several locations in the plane of the diffraction grating were measured, and approximately the same shape was obtained. The representative results are shown in Figure 7. The maximum depth of the structures was around 350 nm. Because the diffraction grating has a dual period, the difference in spatial frequency is observed from the microscope image. From these measurements, two pitches are estimated. The averaged pitch derived from the profile by the AFM measurement has an average value of two pitches and was measured to be approximately 1.023 µm. From the image taken by microscope, the difference between periods of grating was about 46 μm, which is a beat period. Given this average value of the period from AFM measurements and the value of the pitch from microscopy, the two pitches were calculated using the following equations.

(6) $${\Lambda_{ave}}^{-1}=\frac{1}{2}\left({\Lambda_{1}}^{-1}+{\Lambda_{2}}^{-1}\right)$$

(7) $${\Lambda_{beat}}^{-1}={\Lambda_{1}}^{-1}-{\Lambda_{2}}^{-1}$$

Consequently, the spatial frequencies were 0.966 μm^−1^ (1035 nm) and 0.988 μm^−1^ (1012 nm). These have a spatial frequency difference of 0.022 µm^−1^ and satisfy the designed performance. The diffraction efficiency of the fabricated dual-periodic grating for a 45° angle of incidence was measured to be 67.7% and 13.4% for zero-order and first-order light, respectively, using a power meter at a wavelength of 1600 nm. The efficiency of first-order diffracted light was calculated by summing the amount of diffracted lights from the two periods.

## 5. Experimental Validation of Longitudinal Mode Number Estimation

### 5.1. Characteristic of Optical Resonance in Two Frequency Bands

**Figure 8:**
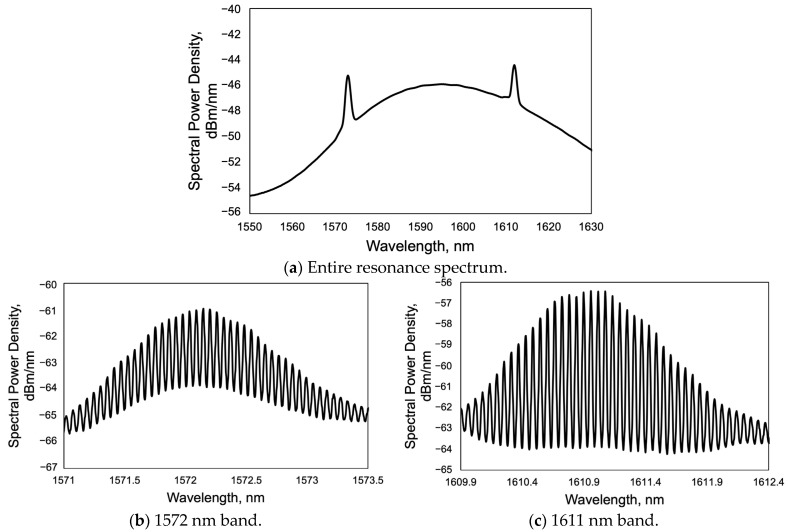
Resonance wavelength spectrum of ECDL measured by optical spectrum analyzer.

The resonance states were measured using the experimental setup described in Chapter 4 and a dual periodic grating. The spectrum of the laser was measured, and the results are shown in Figure 8a. The wavelength of the light source can be observed in the range from approximately 1570 nm to 1630 nm, centered at 1590 nm. The resonance phenomena in the two wavelength bands were confirmed at approximately 1572 nm and 1611 nm. The difference between their resonance wavelengths is approximately 39 nm, which is close to the design value. The width of each resonance wavelength was approximately 2 nm. Although the angle of incidence was set at about 50 degrees, the incident angle was considered to be 51 degrees. The resonant wavelengths calculated by the grating equation were 1573 nm and 1609 nm. This means the measured resonance was theoretically validated. The subtle error was considered to be a measurement error in pitch. For each peak, the measurement range was limited, and the detailed observations are shown in Figure 8b,c. Longitudinal modes were clearly observed when the image was magnified in each resonance wavelength band. About 40 modes were oscillating in each resonance wavelength band. The output resolution of the optical spacer is 20 pm. To estimate the resonance peak with higher resolution, these resonance wavelengths were estimated from the top of each mode by quadratic fitting. The resonant wavelength was measured 10 times at intervals of several seconds under the same conditions, and the standard deviation was about 1.5 pm. The full width half maximum (FWHM) of each mode was approximately 24 pm.

### 5.2. Longitudinal Mode Number Estimation

**Figure 9:**
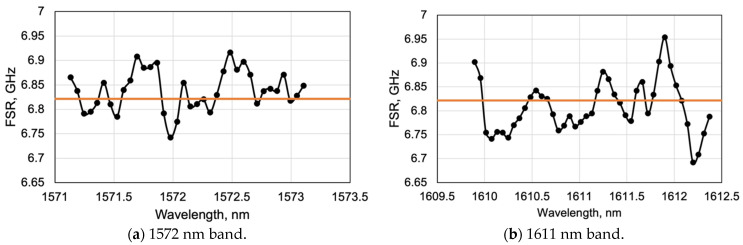
Free spectral range of the resonated modes. The black dots and lines show the experimental values, and the orange line shows the average value of 6.82 Hz.

The longitudinal mode numbers were estimated by measuring the resonant wavelengths. Two resonant modes were to be selected. Then, the mode number difference k of the resonant modes was determined. For example, if the modes at close wavelengths are selected, the mode number difference should just be counted. However, if the modes are compared in distant wavelength bands, as shown in Figure 8b,c, the mode number difference cannot be counted. Therefore, it was considered the free spectrum range (FSR). In the case of considering the resonant modes in the frequency domain, the frequency difference between the modes should be equally spaced, so the frequency difference between the two modes divided by the FSR is expected to be the mode number difference k. In the frequency domain, the spacing between resonant modes is constant, making it suitable for comparing the mode spacing. Figure 9 shows the distribution of the FSR in two resonant wavelength bands based on the data shown in Figure 8b,c. The average value of FSR, which was 6.82 GHz, is represented by the orange line. The FSR was distributed by approximately 0.1 GHz. From the above, k can be obtained using the FSR, and the frequency of each mode is as follows.

(8) $$k=round\left(\frac{\nu_{m}-\nu_{m+k}}{FSR}\right)$$

Using the measured λ_m_, λ_m+k_, and estimated k, the mode number m was estimated from Equation (2). The refractive index n of the medium was supposed to be n^2^ = A + Bλ^2^/(λ^2^ − C^2^), where A = 7.255, B = 2.316, C^2^ = 0.3922 × 10^8^, and here λ was expressed in angstroms [34]. Finally, the resonator length L was obtained from Equation (1). The refractive index of air was compensated using the Ciddor’s equation [35].

As experimental validation, the resonant wavelength was measured eight times at intervals of several seconds under the same conditions. The wavelengths of the two wavelengths were 1.571 133 2 μm and 1.612 373 6 μm, respectively, so the mode number difference k was calculated to be 717. Mode number m was measured with a mean of approximately 28,178 for 1.571 μm and 27,462 for 1.612 μm. These results are summarized in Table 1. The results in Table 1 show that a difference of around 7.8 µm occurs in the L measurement. This error was derived from the refractive index dispersion of the laser gain chip medium, which was assumed to be indium phosphide (InP) in this case. However, it seemed that the difference in dispersion affected errors in the mode number estimation. Higher measurement accuracy can be achieved by compensating for the refractive index of the laser medium [36].

### 5.3. Discussion

The mode number could be determined using the dual periodic grating. In order to verify the proposed method, a variation in mode number estimation by changing the mode number difference k was conducted. The longitudinal mode number was estimated using Equation (1) from all available combinations that account for the mode number difference. When an arbitrary mode is set as m, the average of the estimated longitudinal mode number and the variation in the estimated mode number, *dm*_(k)_, were calculated.

(9) $${dm}_{\left(k\right)}=\sqrt{\frac{1}{N}\sum_{i=0}^{N-1}{\left(m_{\left(\lambda_{m+i},\;\lambda_{m+i+k}\right)}-\bar{m}\right)}^{2}}$$

Here, the resonant wavelength of 1.612 430 537 µm was set as the longitudinal mode number m. This operation was performed for each of the mode number differences, k, ranging from 1 to 40. As in the case of the dual periodic diffraction grating, from all combinations that account for the mode number difference, k, range from 672 to 717. The number of available combinations depends on the value of k. For example, when k was 1, 25, or 42, two modes were picked from one wavelength band as shown in Figure 8b, and their number of combinations was 43, 19, or 2. Similarly, when k was 675 and 714 taken from two wavelength bands as shown in Figure 8b,c, the number of combinations was 23 and 5, respectively. Statistical quantities differed; however, they were still meaningful for discussing the variability of measurements.

**Figure 10:**
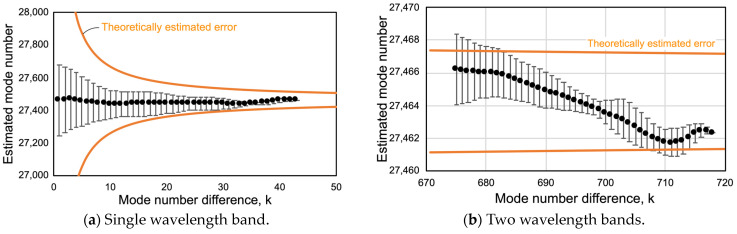
Variation in longitudinal mode number estimation depending on the mode number difference. The black dots and lines indicate experimental values, and the solid orange line indicates the theoretical estimated error when the wavelength measurement error is assumed to be 3 pm.

The results are shown in Figure 10. The horizontal axis is the mode number difference, and the vertical axis is the standard deviation of the estimated mode number, m, for each combination. Figure 10a shows the case where two modes are selected in one wavelength band shown in Figure 8b, while Figure 10b shows the case where two modes are selected across two wavelength bands shown in Figure 8b,c. This means that the mode number difference improved roughly by a factor of 20 by using the dual periodic grating. The orange solid line shows the mode number estimation error assuming a wavelength measurement error of 3 pm. In Figure 10a, the standard deviation of the mode number variation was approximately 10 to a few 100, whereas in Figure 10b, the standard deviation was less than 10. When k was 717, the mean value was 27,362.4 and the standard deviation was 0.39. Thus, by using the dual periodic grating, it was possible to obtain a large mode number difference and to confirm the improved accuracy of the mode number estimation. As discussed in Section 3.2, in optical profiler applications, the accuracy of mode number estimation has the greatest impact on measurement sensitivity. The uncertainty of the sensitivity can be calculated using Equation (4), i.e., dm/m = 0.39/27,462.4 = 1.4 × 10^−5^. This is expected to be sufficiently high accuracy in optical profilers.

In this case, the mode number difference, k, could be increased to around 700. From Figure 3, increasing the mode number difference k further does not significantly improve the mode number estimation error. On the other hand, a large improvement can be seen by reducing the measurement error of the resonance wavelength. A measurement error of about 0.1 pm in wavelength is sufficient to uniquely estimate the mode number under the present measurement conditions. In this case, though, the optical spectrum analyzer was used to verify the principle, if FSR and wavelength measurements could be carried out by beat signal acquisition using a standard laser source such as an acetylene laser, the accuracy would be further improved, and the measurement speed would also be significantly increased.

## 6. Conclusions

In this paper, we proposed an external cavity diode laser-based optical profiler system for dimensional measurement. A crucial aspect of this system is the technique used to determine the mode number. Numerical analysis revealed that to accurately estimate the mode number, the difference between the mode numbers used in the calculation should be sufficiently large. Additionally, the importance of improving the accuracy of resonance wavelength measurements was emphasized. In our experiments, mode number estimation using a conventional diffraction grating showed that a mode number difference of 40 resulted in a variation in mode number estimation of around 10, with a relative accuracy of 3.6 × 10^−4^. However, when using a dual-periodic grating, the mode number difference could be increased to around 700. In the proposed method, the variation in mode number estimation improved to 0.39, yielding a relative accuracy of 1.4 × 10^−5^, which is more than one order of magnitude better than the conventional method. This confirmed the significant improvement in mode number estimation accuracy achieved by using the dual-periodic grating.

For even higher accuracy in estimation, it is essential to consider the refractive index dispersion of the laser gain medium. To achieve this, improving the accuracy of resonance wavelength measurement is crucial. This could be accomplished by using wavelength-stabilized lasers, such as acetylene lasers or optical frequency combs.

## Figures and Tables

**Table 1:** Measured results.

	Mode	
	m+k	m
Resonant wavelength, μm	1.571 133 2	1.612 373 6
Resonant frequency, THz	190.812 878 7	185.932 383 0
FSR (averaged), GHz	6.8215	
Mode number difference, k	716	
Estimated mode number	28,178	27,462
Estimated cavity length, L, mm	18.966 89	18.974 81

## Data Availability

The data that support the findings of this study are available from the corresponding author upon reasonable request.
